# Comparison of the expression of cluster of differentiation (CD)39 and CD73 between propofol- and sevoflurane-based anaesthesia during open heart surgery

**DOI:** 10.1038/s41598-018-28505-8

**Published:** 2018-07-05

**Authors:** Chung-Sik Oh, Karam Kim, Woon-Seok Kang, Nam-Sik Woo, Po-Soon Kang, Jun-Seok Kim, Hang-Rae Kim, Seung-Hyun Lee, Seong-Hyop Kim

**Affiliations:** 1 Department of Anaesthesiology and Pain Medicine, Konkuk University Medical Centre, Konkuk University School of Medicine, Seoul, Korea; 2 Department of Anaesthesiology and Pain Medicine, Konyang University Hospital, Konyang University College of Medicine, Daejeon, Korea; 3 Department of Thoracic and Cardiovascular Surgery, Konkuk University Medical Centre, Konkuk University School of Medicine, Seoul, Korea; 4 Department of Anatomy and Cell Biology, Seoul National University College of Medicine, Seoul, South Korea; 5 Department of Microbiology, Konkuk University School of Medicine, Seoul, Korea; 6 Department of Medicine, Institute of Biomedical Science and Technology, Konkuk University School of Medicine, Seoul, Korea; 7 Department of Infection and Immunology, Konkuk University School of Medicine, Seoul, Korea

## Abstract

High expression of cluster of differentiation (CD)39 and CD73 has cardio-protective effects. We hypothesised that the expression of CD39 and CD73 would differ between propofol- and volatile anaesthetic-based anaesthesia in patients undergoing open heart surgery (OHS). The objective of this prospective randomized trial was to compare the changes in CD39 and CD73 levels in CD4^+^ T cells between propofol- and sevoflurane-based anaesthesia during OHS. The study randomly allocated 156 patients undergoing OHS to a propofol or sevoflurane group. Blood was obtained preoperatively and up to 48 hours after weaning from cardiopulmonary bypass (CPB). The expression levels of CD39 and CD73 in circulating CD4^+^ T cells, serum cytokines and other laboratory parameters were analysed. The primary outcome was the expression of CD39 and CD73 on CD4^+^ T cells. Demographic data and perioperative haemodynamic changes did not show significant differences between the two groups. The expression of CD39 and CD73 in the sevoflurane group was significantly lower than in the propofol group (*P* < 0.001). Other laboratory findings including cardiac enzymes and cytokine levels, did not show significant intergroup differences. Propofol attenuated the decrease in CD39 and CD73 in circulating CD4^+^ T cells compared to sevoflurane-based anaesthesia during OHS.

## Introduction

Open heart surgery (OHS) with aortic cross clamping induces ischemia-reperfusion injury (IRI) accompanied by a systemic inflammatory response, resulting in critical postoperative complications^1–3^.

Propofol and volatile anaesthetics are the most popular agents for OHS, and propofol may be a useful organ-protective anaesthetic due to its anti-inflammatory properties^4,5^. In addition, volatile anaesthetics may have organ-protective properties through their preconditioning effect^6^. The choice of anaesthetic for OHS is important to minimise inflammatory responses and related postoperative complications. However, the most favourable anaesthetics during OHS have not been identified^7,8^.

Cluster of differentiation (CD)39 and CD73 contribute to adenosine formation^9^, where adenosine shifts the pro-inflammatory role of adenosine triphosphate (ATP) towards an anti-inflammatory role^10^. The cardiac protective roles of CD39 and CD73 against IRI have been demonstrated in previous studies^11,12^. However, a comparison of the change in CD39 and CD73 expression between propofol- and volatile anaesthetic-based anaesthesia during OHS has not been conducted. Therefore, characterising the responses of CD39 and CD73 in patients anaesthetised by propofol or volatile anaesthetics during OHS could increase our understanding on the mechanism underlying the anti-inflammatory effect of propofol and volatile anaesthetics against IRI during OHS.

We hypothesised that the expression of CD39 and CD73 would differ between propofol- and volatile anaesthetic-based anaesthesia in patients undergoing OHS. This study was designed to investigate the expression CD39 and CD73 in the circulating CD4^+^ T cells of patients undergoing OHS under propofol- and sevoflurane-based anaesthesia.

## Results

**Figure 1:**
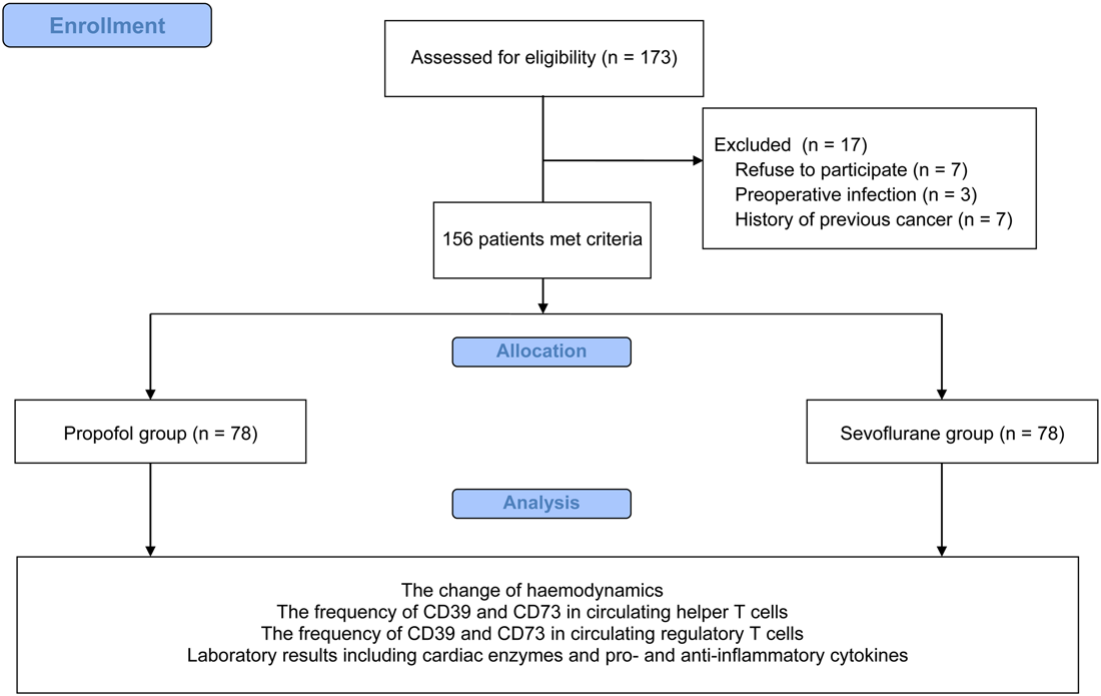
CONSORT diagram.

In total, 173 patients from May 2014 to December 2016 were eligible for the study. A total of 17 patients were excluded for the following reasons: 7 refused to participate, 3 had a preoperative infection, and 7 had a previous history of cancer, and there were no adverse events during the study. Therefore, 156 patients were included in the final analysis (78 in the propofol group and 78 in the sevoflurane group) (Fig. 1). The patient demographics were similar between the propofol and sevoflurane groups (Table 1). Perioperative haemodynamic changes up to 48 hours after weaning from CPB were also similar between the two groups (Table 2).

**Table 1:** Patient demographic data.

	Propofol group (n = 78)	Sevoflurane group (n = 78)	*P*	
Sex	Male/female	40/38	30/48	0.147
Age (years)		54.0 (45.0–64.0)	58.7 (41.0–71.0)	0.428
Height (cm)		162.8 ± 10.0	162.5 ± 6.8	0.825
Weight (kg)		62.5 (54.8–68.9)	57.0 (55.7–70.0)	0.644
Underlying disease				
	Hypertension	28 (35.9%)	34 (43.6%)	0.413
	Diabetes mellitus	7 (9.0%)	5 (6.4%)	0.764
	Arrhythmia	17 (21.8%)	10 (12.8%)	0.204
	CVA	5 (6.4%)	3 (3.8%)	0.717
Type of operation				0.231
	AVP	37 (47.4%)	36 (46.2%)	
	AVR	3 (3.8%)	9 (11.5%)	
	MVP	20 (25.6%)	16 (20.5%)	
	PVR	4 (5.1%)	6 (7.7%)	
	TAP	3 (3.8%)	0 (0.0%)	
	DVP	11 (14.1%)	11 (14.1%)	
Preoperative LV ejection fraction (%)		65.0 (60.9–68.0)	63.4 (59.0–71.6)	0.584
Anaesthetics				
	Propofol (mg)	994.0 (985.0–1024.0)	0.0 (0.0–0.0)	0.000
	Remifentanil (μg)	6289.0 (4819.0–8164.0)	6256.5 (4315.0–8264.0)	0.398
Fluid requirements				
	Crystalloid (ml)	1400.0 (1300.0–1800.0)	1400.0 (1300.0–1900.0)	0.741
	Colloid (ml)	600.0 (500.0–700.0)	600.0 (500.0–600.0)	0.138
Duration of anaesthesia (min)		410.0 (350.0–475.0)	401.5 (360.0–456.0)	0.348
Duration of operation (min)		335.0 (285.0–400.0)	335.0 (290.0–365.0)	0.932
Duration of cardiopulmonary bypass (min)		164.5 (132.0–198.0)	168.0 (145.0–227.0)	0.407
Duration of aortic cross clamp (min)		108.0 (79.0–137.0)	109.0 (102.0–149.0)	0.256
Duration of mechanical ventilation (min)		1643.0 (1400.0–1894.0)	1605.0 (1505.0–2098.0)	0.424
Duration of ICU stay (min)		4090.5 (2810.0–4325.0)	4065.0 (2780.0–5106.0)	0.932
Duration of hospital stay (days)		19.0 (16.0–22.0)	20.0 (17.0–22.0)	0.362
Previous medications				
	ARB	23 (29.5%)	28 (35.9%)	0.495
	CCB	13 (16.7%)	8 (10.3%)	0.348
	Beta blocker	3 (3.8%)	5 (6.4%)	0.717
	Nitrate	9 (11.5%)	11 (14.1%)	0.811
	Aspirin	19 (24.4%)	17 (21.8%)	0.849
	Statin	5 (6.4%)	5 (6.4%)	1.000
Type of valvular disease				0.648
	AR	18 (23.1%)	19 (24.4%)	
	AS	22 (28.2%)	26 (33.3%)	
	MR	16 (20.5%)	13 (16.7%)	
	MS	4 (5.1%)	3 (3.8%)	
	PR	4 (5.1%)	6 (7.7%)	
	TR	3 (3.8%)	0 (0.0%)	
	DVD	11 (14.1%)	11 (14.1%)	
Perioperative drugs				
	Dopamine (mg)	41.6 (17.6–63.9)	31.7 (15.2–45.9)	0.142
	Dobutamine (mg)	0.0 (0.0–11.2)	0.0 (0.0–12.1)	0.526
	Milrinone (mg)	0.0 (0.0–0.9)	0.0 (0.0–0.6)	0.259
	Phenylephrine (mg)	0.6 (0.0–1.5)	0.5 (0.0–1.2)	0.370
	Norepinephrine (mg)	0.0 (0.0–0.0)	0.0 (0.0–0.0)	0.652
	Isoproterenol (mg)	0.0 (0.0–0.0)	0.0 (0.0–0.0)	0.388
	Nitroglycerine (mg)	0.0 (0.0–0.0)	0.0 (0.0–0.0)	0.796
Transfusion requirements				
	pRBC (units)	4.0 (2.0–6.0)	5.0 (3.0–6.0)	0.095
	FFP (units)	1.5 (0.0–2.0)	1.0 (0.0–2.0)	0.557
	PC (units)			0.607
	8	41 (52.6%)	46 (59.0%)	
	16	9 (11.5%)	8 (10.3%)	
	32	0 (0.0%)	1 (1.3%)	
	Cryoprecipitate (units)			0.287
	8	7 (9.0%)	7 (9.0%)	
	10	5 (6.4%)	5 (6.4%)	
	16	1 (1.3%)	6 (7.7%)	

Data are expressed as numbers (percentages), median values (25–75%), or means ± standard deviation.

Abbreviations: CVA, cerebrovascular attack; AVP, aortic valvuloplasty; AVR, aortic valve replacement; MVP, mitral valvuloplasty; MVR, mitral valve replacement; PVR, pulmonary valve replacement; TAP, tricuspid annuloplasty; DVP, double valvuloplasty; Perioperative drugs, total drug dosage used from start of anaesthesia to 48 hours after weaning from cardiopulmonary bypass; ICU, intensive care unit; AR, aortic regurgitation; AS, aortic stenosis; MR, mitral regurgitation; MS, mitral stenosis; PR, pulmonary regurgitation; TR, tricuspid regurgitation; DVD, double valvular disease; AVP, aortic valvuloplasty; AVR, aortic valve replacement; MVP, mitral valvuloplasty; MVR, mitral valve replacement; PVR, pulmonary valve replacement; TAP, tricuspid annuloplasty; DVP, double valvuloplasty; pRBC, packed red blood cell; FFP, fresh frozen plasma; PC, platelet concentration.

**Table 2:** Haemodynamic changes during open heart surgery.

	Propofol group (n = 78)	Sevoflurane group (n = 78)	*P*
Mean BP (mmHg)			
Preop	83.5 (77.7–93.3)	86.3 (75.0–90.3)	0.816
Weaning	72.8 (68.3–78.0)	73.3 (61.7–78.3)	0.621
3 hours	84.0 ± 10.8	82.7 ± 10.8	0.478
24 hours	87.6 ± 9.4	86.8 ± 10.6	0.615
48 hours	90.9 ± 9.0	90.8 ± 10.2	0.901
HR (beats/min)			
Preop	75.0 (69.0–85.0)	79.0 (71.0–85.0)	0.140
Weaning	79.0 (72.0–85.0)	76.0 (68.0–85.0)	0.377
3 hours	82.0 (76.0–88.0)	81.0 (76.0–87.0)	0.975
24 hours	76.0 (72.0–84.0)	77.0 (72.0–90.0)	0.279
48 hours	78.5 (75.0–87.0)	79.0 (74.0–92.0)	0.328
CVP (mmHg)			
Preop	7.0 (5.0–10.0)	7.0 (6.0–9.0)	0.619
Weaning	9.0 (7.0–11.0)	9.0 (8.0–10.0)	0.378
3 hours	9.0 (7.0–11.0)	9.0 (8.0–10.0)	0.339
24 hours	9.0 (8.0–11.0)	9.0 (8.0–11.0)	0.358
48 hours	8.0 (6.0–9.0)	8.0 (6.0–10.0)	0.953
CI (l/min/m^2^)			
Preop	2.5 (2.2–2.8)	2.3 (2.1–3.1)	0.826
Weaning	2.4 (2.1–2.9)	2.4 (2.2–2.7)	0.949
3 hours	2.7 ± 0.6	2.6 ± 0.4	0.429
24 hours	2.8 (2.5–3.0)	2.8 (2.6–3.1)	0.551
48 hours	2.8 (2.6–3.1)	2.7 (2.6–3.0)	0.244

Data are expressed as median values (25–75%) or means ± standard deviation.

Abbreviations: BP, blood pressure; Preop, preoperative time; Weaning, immediate after weaning from cardiopulmonary bypass (CPB); 3 hours, 3 hours after weaning from CPB; 24 hours, 24 hours after weaning from CPB; 48 hours, 48 hours after weaning from CPB; HR, heart rate; CVP, central venous pressure; CI, cardiac index.

### Expression of CD39 and CD73 in circulating helper T cells during OHS

The expression of CD39 in circulating CD4^+^ T cells was lowest 3 hours after weaning from CPB and increased with time (Supplementary Fig. 1). The expression of CD73 in circulating CD4^+^ T cells was lowest immediately after weaning from CPB and increased with time (Supplementary Fig. 2).

### Expression of CD39 and CD73 in circulating helper T cells between propofol- and sevoflurane-based anaesthesia during OHS

**Figure 2:**
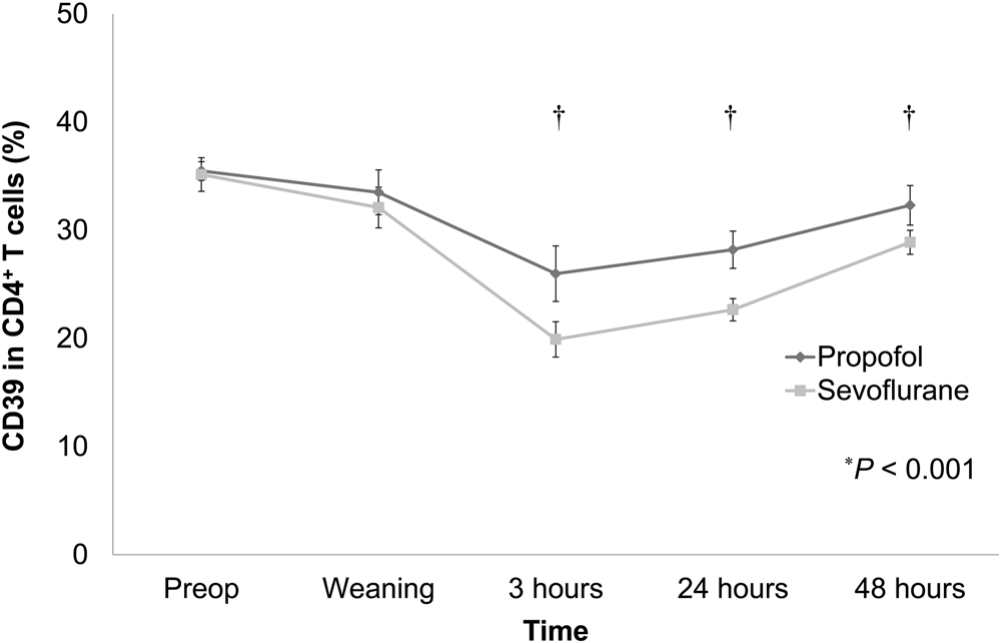
Comparison of changes in CD39 expression in circulating helper T cells between the propofol- and sevoflurane-based anaesthesia groups during open heart surgery (OHS). The expression of CD39 was significantly lower in the sevoflurane group. ^*^An overall significant difference between the propofol and sevoflurane groups. ^†^Pairwise comparison (*P* < 0.05) at each time point with Bonferroni’s correction. Abbreviations: Preop, preoperative time; Weaning, immediate after weaning from cardiopulmonary bypass (CPB); 3 hours, 3 hours after weaning from CPB; 24 hours, 24 hours after weaning from CPB; 48 hours, 48 hours after weaning from CPB.

**Figure 3:**
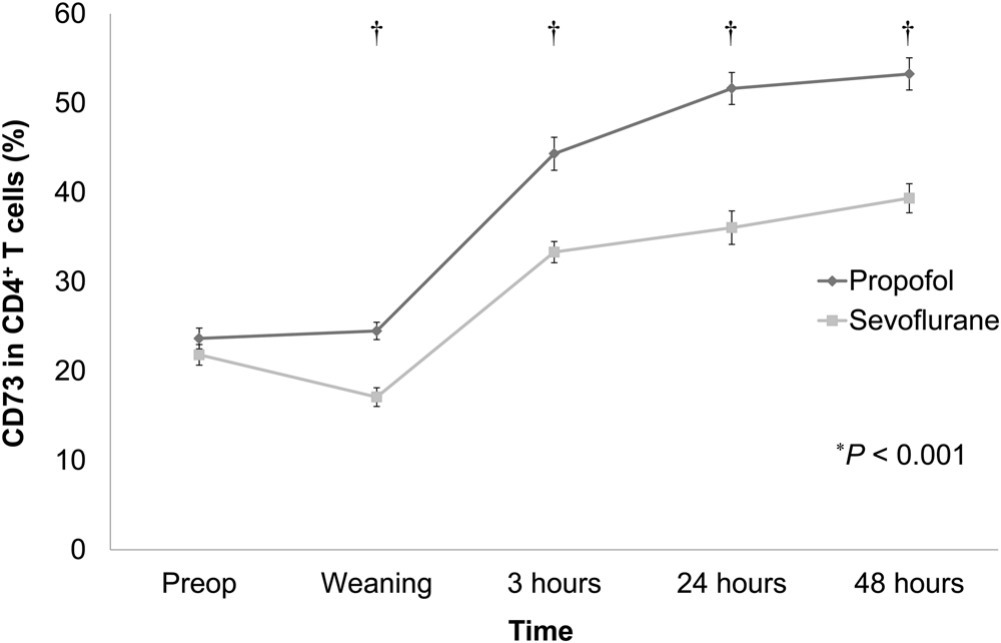
Comparison of changes in CD73 expression in circulating helper T cells between the propofol- and sevoflurane-based anaesthesia groups during OHS. The expression of CD73 was significantly lower in the sevoflurane group. ^*^An overall significant difference between the propofol and sevoflurane groups. ^†^Pairwise comparison (*P* < 0.05) at each time point with Bonferroni’s correction. Abbreviations: Preop, preoperative time; Weaning, immediate after weaning from CPB; 3 hours, 3 hours after weaning from CPB; 24 hours, 24 hours after weaning from CPB; 48 hours, 48 hours after weaning from CPB.

The overall change in CD39 expression in circulating CD4^+^ T cells was significantly lower in the sevoflurane group than in the propofol group (*P* < 0.001) (Fig. 2). Especially, the expression of CD39 in circulating CD4^+^ T cells was significantly lower from 3 hours until 48 hours after weaning from CPB in the sevoflurane group with Bonferroni’s correction (Fig. 2). The overall change in CD73 expression in circulating CD4^+^ T cells was also significantly lower in the sevoflurane group than in the propofol group (*P* < 0.001) (Fig. 3). Especially, the expression of CD73 in circulating CD4^+^ T cells from immediately after up to 48 hours after weaning from CPB was significantly lower in the sevoflurane group with Bonferroni’s correction (Fig. 3).

### Expression of CD39 and CD73 in circulating regulatory T cells during OHS

The pattern of CD39 and CD73 expression in circulating CD4^+^CD25^+^Foxp3^+^ T cells was similar to the pattern in CD4^+^ T cells. The expression of CD39 in circulating CD4^+^CD25^+^Foxp3^+^ T cells was lowest 3 hours after weaning from CPB and increased with time (Supplementary Fig. 3). The expression of CD73 in circulating CD4^+^CD25^+^Foxp3^+^ T cells was lowest immediately after weaning from CPB and increased with time (Supplementary Fig. 4).

### Expression of CD39 and CD73 in circulating regulatory T cells in propofol- and sevoflurane-based anaesthesia during OHS

The expression of CD39 in circulating CD4^+^CD25^+^Foxp3^+^ T cells was lower in the sevoflurane group than in the propofol group. The expression of CD73 in circulating CD4^+^CD25^+^Foxp3^+^ T cells was also lower in the sevoflurane group. However, the overall change in CD39 and CD73 expression in circulating CD4^+^CD25^+^Foxp3^+^ T cells did not differ significantly between the groups (*P* = 0.161 for CD39 and *P* = 0.068 for CD73) (Supplementary Figs 5 and 6).

### Mean fluorescence intensity of CD39 and CD73 in circulating helper T cells and regulatory T cells

The patterns of the mean fluorescence intensity of CD39 and CD73 in circulating helper T cells and regulatory T cells were similar to the results regarding frequency (Supplementary Table 1).

### Changes in laboratory results and cytokines with propofol- versus sevoflurane-based anaesthesia

The laboratory results did not differ between the two groups (Table 3). No pro- or anti-inflammatory cytokines, such as interleukin (IL)−1, IL-6, IL-10, IL-17, interferon (IFN)-γ, and tumour necrosis factor (TNF)-α, showed significant intergroup differences (Table 4).

**Table 3:** Changes in laboratory results during open heart surgery.

	Propofol group (n = 78)	Sevoflurane group (n = 78)	*P*
WBC (/μl)			
Preop	6395 (5480–7260)	6523 (5710–7670)	0.144
3 hours	8995 (6280–10950)	9715 (7020–11532)	0.405
24 hours	11240 (9550–13500)	11250 (9365–12545)	0.850
48 hours	11740 (10000–14000)	11730 (9845–13025)	0.897
Neutrophils (%)			
Preop	52.8 ± 10.2	52.0 ± 9.4	0.639
3 hours	86.4 (81.9–88.7)	85.9 (84.2–88.4)	0.674
24 hours	88.3 (84.3–90.5)	86.0 (83.4–90.3)	0.216
48 hours	89.0 (85.3–91.4)	87.0 (84.2–91.1)	0.306
Lymphocytes (%)			
Preop	34.6 (28.9–41.3)	34.1 (31.1–40.5)	0.844
3 hours	8.9 (5.6–12.0)	7.7 (5.8–10.9)	0.516
24 hours	4.3 (3.3–5.5)	4.3 (3.7–4.7)	0.585
48 hours	6.5 (5.7–7.9)	6.4 (5.9–7.1)	0.633
ESR (mm/hour)			
Preop	10.0 (3.0–16.0)	9.0 (7.0–18.0)	0.128
3 hours	2.0 (2.0–2.0)	2.0 (2.0–2.0)	0.220
24 hours	2.0 (2.0–5.0)	2.5 (2.0–4.0)	0.610
48 hours	2.0 (1.0–4.0)	2.0 (1.0–3.0)	0.821
hs-CRP (mg/dl)			
Preop	0.3 (0.2–0.4)	0.3 (0.3–0.3)	0.386
3 hours	0.2 (0.1–0.3)	0.2 (0.1–0.3)	0.101
24 hours	4.5 (3.2–6.0)	4.4 (3.1–6.1)	0.852
48 hours	5.0 (3.7–6.5)	4.7 (3.4–6.4)	0.565
CK-MB (ng/ml)			
Preop	1.1 (0.7–1.8)	1.1 (1.0–2.0)	0.376
3 hours	36.0 (28.3–42.5)	35.5 (32.1–44.9)	0.557
24 hours	23.1 (16.8–27.0)	21.6 (16.2–29.6)	0.468
48 hours	21.0 (14.7–25.0)	18.6 (13.2–26.6)	0.239
hs-TnI (ng/l)			
Preop	0.0 (0.0–0.0)	0.0 (0.0–0.0)	0.836
3 hours	9.4 (4.6–14.5)	6.4 (5.1–8.6)	0.139
24 hours	8.8 (3.9–22.8)	9.6 (7.8–11.1)	0.093
48 hours	7.4 (2.4–21.3)	8.1 (6.4–9.8)	0.122
NT-pro BNP (pg/ml)			
Preop	156.1 (40.9–257.2)	82.3 (34.2–324.0)	0.449
3 hours	141.4 (62.9–236.0)	176.8 (109.1–235.0)	0.151
24 hours	235.8 (155.9–295.0)	221.6 (165.0–358.0)	0.246
48 hours	285.8 (203.9–347.0)	276.6 (217.0–412.0)	0.199
PF ratio			
Preop	486.8 (447.1–517.1)	502.9 (444.2–529.5)	0.563
Weaning	264.2 (127.5–407.0)	297.2 (185.7–375.0)	0.299
3 hours	359.3 (267.7–437.5)	350.9 (259.8–437.5)	0.975
24 hours	363.8 (304.0–434.0)	357.8 (300.0–417.5)	0.884
48 hours	513.7 (428.6–646.2)	493.7 (416.7–630.0)	0.655
Hct (%)			
Preop	38.4 (35.7–41.7)	38.7 (37.1–39.5)	0.630
Weaning	30.5 (29.0–32.0)	31.0 (28.0–32.0)	0.471
3 hours	32.4 (29.7–34.9)	32.3 (30.0–33.1)	0.489
24 hours	36.5 (34.6–38.3)	36.3 (33.1–37.6)	0.435
48 hours	37.7 (35.8–39.5)	37.5 (34.2–38.8)	0.391
pH			
Preop	7.4 (7.4–7.5)	7.4 (7.4–7.5)	0.928
Weaning	7.4 ± 0.0	7.4 ± 0.0	0.376
3 hours	7.4 (7.4–7.5)	7.4 (7.4–7.5)	0.406
24 hours	7.4 (7.4–7.5)	7.4 (7.4–7.5)	0.412
48 hours	7.4 (7.4–7.5)	7.5 (7.4–7.5)	0.310
Lactate (mmol/l)			
Preop	1.5 (1.4–1.7)	1.5 (1.4–1.6)	0.211
Weaning	2.2 (1.7–2.9)	2.5 (1.6–3.4)	0.387
3 hours	2.5 (1.9–3.0)	2.4 (1.8–3.1)	0.721
24 hours	1.8 (1.4–2.6)	2.2 (1.3–2.7)	0.638
48 hours	1.7 (1.3–2.5)	2.0 (1.2–2.5)	0.737

Data are expressed as median values (25–75%) or means ± standard deviation. **Abbreviations:** WBC, white blood cell; Preop, preoperative time; 3 hours, 3 hours after weaning from cardiopulmonary bypass (CPB), 24 hours, 24 hours after weaning from CPB; 48 hours, 48 hours after weaning from CPB; Weaning, immediate after weaning from CPB; ESR, erythrocyte sedimentation rate; hs-CRP, high-sensitivity; C- reactive protein; CK-MB, creatine kinase-MB; hs-TnI, high-sensitivity troponin (Tn)I; NT-pro BNP, N-terminal-pro brain natriuretic peptide; PF ratio, the ratio of arterial oxygen partial pressure to fractional inspired oxygen; Hct, haematocrit.

**Table 4:** Changes in cytokine levels during open heart surgery.

	Propofol group (n = 78)	Sevoflurane group (n = 78)	*P*
IL-1 (%)			
Preop	1.0 (0.5–1.4)	1.1 (0.6–1.2)	0.687
Weaning	1.6 (0.8–1.9)	1.6 (0.9–2.4)	0.257
3 hours	0.9 (0.5–1.6)	0.9 (0.5–1.5)	0.622
24 hours	0.6 (0.4–1.0)	0.5 (0.3–1.0)	0.759
48 hours	0.5 (0.2–0.9)	0.4 (0.2–0.9)	0.973
IL-6 (%)			
Preop	1.7 (0.7–1.9)	1.7 (0.9–2.0)	0.535
Weaning	1.9 (1.3–2.0)	2.4 (1.5–2.6)	0.050
3 hours	2.2 (0.9–3.7)	1.8 (1.2–3.8)	0.894
24 hours	2.1 (1.0–2.8)	1.6 (0.5–4.0)	0.296
48 hours	2.1 (0.9–2.7)	1.5 (0.5–4.0)	0.318
IL-10 (%)			
Preop	2.1 (0.7–3.4)	2.3 (1.4–4.4)	0.425
Weaning	1.3 (0.6–2.7)	1.3 (0.8–2.4)	0.880
3 hours	1.0 (0.4–2.7)	1.2 (0.8–1.7)	0.287
24 hours	2.7 (1.4–3.8)	3.3 (1.1–4.0)	0.511
48 hours	2.6 (1.3–3.6)	3.2 (0.9–3.9)	0.539
IL-17 (%)			
Preop	1.3 (0.5–2.3)	1.4 (0.3–2.4)	0.444
Weaning	3.7 (2.8–5.1)	3.7 (2.9–4.6)	0.353
3 hours	2.1 (1.2–3.1)	2.4 (1.7–3.1)	0.073
24 hours	1.9 (0.8–2.1)	1.4 (0.2–2.2)	0.080
48 hours	1.8 (0.8–2.1)	1.3 (0.1–2.2)	0.134
IFN-γ (%)			
Preop	4.1 (0.8–7.6)	6.7 (3.7–8.3)	0.199
Weaning	12.9 (1.0–20.1)	12.9 (6.7–14.6)	0.928
3 hours	12.6 (1.0–19.9)	13.9 (10.4–15.4)	0.376
24 hours	21.1 (13.1–27.6)	17.1 (9.5–23.4)	0.136
48 hours	22.8 (14.9–29.1)	18.6 (11.1–25.2)	0.130
TNF-α (%)			
Preop	3.3 (1.7–4.1)	3.1 (2.4–3.8)	0.982
Weaning	2.5 (0.3–5.1)	2.9 (0.5–4.8)	0.272
3 hours	3.4 (2.4–4.0)	3.0 (1.2–3.9)	0.153
24 hours	1.5 (0.9–2.0)	1.2 (0.6–3.2)	0.357
48 hours	1.3 (0.8–1.8)	1.1 (0.5–3.1)	0.396

Data are expressed as median values (25–75%) or means ± standard deviation. The cytokine level was the percentage of each cytokine among total CD4^+^ T cells. Abbreviations: IL, interleukin; Preop, preoperative time; Weaning, immediate after weaning from cardiopulmonary bypass (CPB); 3 hours, 3 hours after weaning from CPB, 24 hours, 24 hours after weaning from CPB; 48 hours, 48 hours after weaning from CPB; IFN, interferon; TNF, tumour necrosis factor.

## Discussion

This study showed that the expression of CD39 and CD73 in circulating helper T cells was low immediately after weaning from CPB and recovered over time. In addition, the CD39 and CD73 levels in circulating helper T cells were higher with propofol-based anaesthesia than sevoflurane-based anaesthesia during OHS.

Previous studies showed that CD39 and CD73 have organ-protective effects against IRI^9,13–16^. Kim *et al*. showed that CD73 prevents renal IRI^15^ and Bonner *et al*. showed that up-regulation of CD39 and CD73 confers myocardial protection against cardiac IRI^16^. In addition, blocking CD39 and CD73 could induce organ injury by inhibiting adenosine formation after IRI^17,18^. Meanwhile, OHS involves aortic cross clamping for several hours. Organ blood supply depends on CPB during this ‘myocardial ischemic’ period. After the main surgical procedure, the aortic cross clamp is released and weaning form CPB should follow. Intense IRI related inflammation occurs during this time before and after weaning from CPB^2^. The low expression levels of CD39 and CD73 immediately and 3 hours after weaning from CPB in this study support the notion that this period had the greatest IRI related inflammation during OHS.

Several studies have shown the beneficial effects of volatile anaesthetics relative to propofol during cardiac surgery^19,20^, indexed by lower creatine kinase-MB (CK-MB) levels with volatile anaesthetics compared to propofol-based anaesthesia. However, cardiac enzymes do not guarantee immune status during OHS since they are associated only with myocardial injury related to surgical trauma^21^. In addition, some reports found no differences in cardiac enzyme levels between propofol- and sevoflurane-based anaesthesia during cardiac surgery^22^. Similarly, we found no significant difference in cardiac enzyme and cytokine levels between the two groups. Various perioperative factors, such as the intensity of surgical trauma, haemodynamic changes, transfusion, and drugs, can affect cardiac enzyme and cytokine levels^23–25^ and could be confounding factors. However, our results revealed that immune status, which could not be determined based on cytokine expression alone, could be examined more precisely based on the expression of CD39 and CD73 in helper T cells, even with confounding factors. The decrease in CD39 and CD73 immediately and 3 hours after weaning from CPB was greater in the sevoflurane group. Similarly, the recovery of CD39 and CD73 after weaning from CPB was weaker in the sevoflurane group. In addition, although not significant, IL-6 was higher immediately after weaning from CPB in the sevoflurane group. Because IL-6 can be induced by IRI related inflammation during CPB^3^, we postulated that sevoflurane has a less marked immune regulatory effect against IRI than propofol. Finally, regarding the changes in CD39, CD73, and IL-6, propofol-based anaesthesia might be more beneficial for minimizing IRI related inflammation during OHS. In addition, IRI related inflammation was closely related to postoperative complications after OHS^1^. Therefore, we anticipate potential benefits from propofol use relative to sevoflurane during OHS by reducing IRI related inflammation and postoperative complications.

The clinical outcomes, including the duration of mechanical ventilation and duration of stays in the intensive care unit (ICU) and hospital, did not differ between the groups in our study. Lurati Buse *et al*. found that propofol and sevoflurane had similar impacts on myocardial ischemia and postoperative complications^26^. In addition, a recent large scale meta-analysis found no difference in survival between propofol and volatile anaesthetics^8^. However, enhanced expression of CD39 and CD73 in animal models was recently considered as a novel therapeutic approach to avoid inflammatory responses against IRI^13,14,27–30^. Therefore, regulating the expression of CD39 and CD73 by adjusting the propofol dosage could be a helpful approach for minimizing IRI related inflammation and postoperative complications. Because previous studies of the anti-immunosuppressive effects of CD39 and CD73 used animal models, exploring the effects of CD39 and CD73 according to different anaesthetics may be challenging in the clinical setting.

There could be several considerations in the present study. First, adenosine was not measured directly in the present study. However, the half-life of adenosine is extremely short and it is difficult to measure accurately^31^. Instead, recent studies have focused on blocking the adenosine receptor instead of adenosine itself^10^, or on interventions with several endogenous mediators, such as CD39 and CD73, which are responsible for adenosine formation^13,32^. In this respect, investigating the pattern of CD39 and CD73 expression during OHS may be essential. Second, cytokine production by other leukocyte such as neutrophil and B cells was not measured in the present study. However, CD39 and CD73 are mainly expressed in T cell subpopulation and have main role expressed in T cells. Third, even the patterns of CD39 and CD73 in regulatory T cells were similar in trends of reducing inflammation, there were no statistical significances. Therefore, further clinical investigations about CD39 and CD73 during OHS might help us to understand the exact mechanism of IRI related inflammation during OHS.

In conclusion, the expression of CD39 and CD73 in circulating helper T cells was decreased immediately after weaning from CPB. The decrease in CD39 and CD73 was worse in sevoflurane-based anaesthesia relative to propofol-based anaesthesia. This result may be associated with IRI related inflammation occurring during OHS. Our results suggest that propofol might be better than sevoflurane for reducing IRI related inflammation during OHS.

## Methods

### Study population

This study was approved by the Institutional Review Board of Konkuk University Medical Centre, Seoul, Korea (IRB #KUH1160064) and carried out in accordance to the relevant guidelines and regulation of the Declaration of Helsinki. This study was registered before patient enrollment at clinicaltrials.gov (NCT02136979, Principal investigator: Seong-Hyop, Kim, Date of registration: May 13, 2014) and was conducted at a single tertiary medical centre (Konkuk University Medical Centre). All patients signed a written informed consent. The study used a prospective randomized design and was conducted according to the original protocol from May 2014 to December 2016 (full protocol available on request).

Patients undergoing OHS were enrolled, and patients were excluded if any of the following criteria were met: 1) age < 20 years, 2) pre-operative infection, 3) pre-operative use of an immunosuppressive agent, and 4) previous history of cancer. Patients were randomly assigned to groups by opening sequentially numbered envelopes containing the randomization assignment (third party allocation). The allocation sequence was generated by the clinical research coordination centre in our hospital, which was not otherwise involved in the trial, with random-permuted block randomization conducted using an interactive internet-based response system. The propofol and sevoflurane groups were anaesthetised by propofol and sevoflurane, respectively. All involved anaesthesiologists, surgeons and attending physicians were blinded to the study. All data were collected by trained observers who also were blinded and did not participate in patient care.

### Anaesthetic regimens

The anaesthetic technique was standardised between groups. The patient arrived at the operating room without premedication. After establishing routine invasive systemic arterial blood pressure monitoring and non-invasive patient monitoring (pulse oximetry, electrocardiography, cerebral oximetry, and bispectral index [BIS]), anaesthesia was induced using etomidate 0.2 mg/kg. After confirming loss of consciousness, rocuronium (1.0 mg/kg) was administered. For the propofol group, an initial target concentration at the effect-site of propofol, of 1 μg/ml, was administered using a target-controlled infusion device (Orchestra^®^ Base Primea; Fresenius Vial, Brezins, France). For the sevoflurane group, an initial end-expiratory concentration of sevoflurane 1.5 vol% was administered using a vaporiser (Aladin™; Datex-Ohmeda Division Instrumentarium, Bromma, Sweden). The target concentration of propofol and the end-expiratory concentration of sevoflurane were titrated at 0.1 μg/ml and 0.1 vol%, respectively, to maintain BIS values between 40 and 50. In both groups, remifentanil was gradually administered using a target-controlled infusion device. A target concentration of 10 ng/ml was achieved after 10 min of administration of remifentanil and maintained throughout the procedure. Following induction of anaesthesia, patients were ventilated with 40% oxygen in air. The tidal volume was 6 ml/kg of lean body mass and the respiratory rate was adjusted to maintain the partial pressure of end-tidal carbon dioxide between 35 and 40 mmHg. Additional rocuronium was administered under the guidance of peripheral monitoring of neuromuscular transmission. A pulmonary artery catheter was inserted, and transoesophageal echocardiography was done, after anaesthesia induction.

### Management of haemodynamic changes

Haemodynamic stability was maintained using adequate inotropic and vasoactive agents to ensure that the cardiac index and systemic mean blood pressure were above 2.0 l/min/m and 60 mmHg, respectively. Fluid administration was performed to meet fluid requirements and replace surgically lost blood until the laboratory values met transfusion indications. Perioperative transfusion was performed according to our institutional protocols^33^. After the end of the surgery, the propofol or sevoflurane was stopped and the patient was transferred to the ICU. For the sedation during the transfer, remifentanil 10 ng/ml was maintained and infused continuously for 60 minutes after arrival at the ICU. Decision-making regrading medical treatment was performed in the ICU by the physicians in charge of the unit, who were blinded to the study, based on institutional protocols.^33^

### Blood samples

In all patients, blood was sampled to analyse CD39, CD73, and cytokine expression in circulating CD4^+^ T cells and CD4^+^CD25^+^Foxp3^+^ T cells. Blood samples were obtained before anaesthesia induction and immediately, and 3, 24, and 48 hours after weaning from cardiopulmonary bypass (CPB). Samples were collected in ethylenediaminetetraacetic acid (EDTA) tubes.

### Flow cytometric analysis

Peripheral blood mononuclear cells (PBMCs) were isolated from blood samples using density-gradient centrifugation over a Ficoll-Hypaque gradient (GE Healthcare, Piscataway, NJ, USA). The PBMCs were washed with phosphate-buffered saline (PBS; 137 mM NaCl, 2.7 M KCl, 10 mM Na_2_HPO_4_, 2 mM KH_2_PO_4,_ pH 7.4) and resuspended in Roswell Park Memorial Institute (RPMI) medium 1640 with 1% penicillin and 10% fetal bovine serum (FBS). All flow cytometry data were acquired with a FACS-Aria^TM^ flow cytometer (BD Biosciences, San Jose, CA, USA) and analysed using FlowJo^TM^ software (Tree Star Inc., Ashland, OR, USA).

### Flow cytometric analysis of CD39 and CD73 expression in CD4^+^ T cells

To determine the expression of CD39 and CD73 in circulating CD4^+^ T cells, isolated PBMCs were stained with peridinin chlorophyll protein complex (PerCP)-conjugated anti-human CD4 (BD Biosciences), fluorescein isothiocyanate (FITC)-conjugated anti-human CD39 (BD Biosciences), and phycoerythrin (PE)-cy7-conjugated anti-human CD73 (BD Biosciences). After washing with fluorescence-activated cell sorting (FACS) buffer (0.1% bovine serum albumin (BSA) in PBS), PBMCs were stained for 30 minutes.

### Flow cytometric analysis of CD39 and CD73 expression in CD4^+^CD25^+^Foxp3^+^ T cells

The expression of CD39 and CD73 in circulating CD4^+^CD25^+^Foxp3^+^ T cells, as Tregs, was evaluated using the Human Regulatory T cell Staining Kit (eBioscience, San Diego, CA, USA), according to the manufacturer’s protocol. Single-cell suspensions were incubated with PerCP-conjugated anti-human CD4 (BD Biosciences), allophycocyanin (APC)-conjugated anti-human CD25 (BD Biosciences), FITC-conjugated anti-human CD39 (BD Biosciences), and PE-cy7-conjugated anti-human CD73 (BD Biosciences) antibodies for 30 min in the dark at 4 °C to stain surface-expressed factors.

After washing with flow cytometry staining buffer, the PBMCs were incubated with 1 ml freshly prepared Foxp3 fixation/permeabilization buffer for 20 min at 4 °C in the dark. Then, the cells were washed twice with 2 ml freshly prepared 1 × permeabilization buffer. Next, the cells were stained using a PE-conjugated anti-human Foxp3 (eBioscience) antibody for 30 min in the dark at 4 °C. After washing twice, the number of Foxp3-positive cells in the CD4^+^CD25^+^ cell gating was evaluated by flow cytometry, and the frequency of Foxp3^+^ Treg cells was expressed as a percentage of the total CD4^+^ CD25^+^ cells.

### Flow cytometric analysis of cytokine expression in CD4^+^ T cells

To analyse the cytokine production in T cells, PBMCs were isolated from heparinized venous blood using density-gradient centrifugation over a Ficoll-Hypaque gradient (GE Healthcare). The PBMCs were washed with PBS and resuspended in RPMI 1640 medium with 100 U/mL penicillin, 100 U/mL streptomycin and 10% (v/v) heat-inactivated fetal calf serum (HyClone, Logan, UT, USA). After washing, the cells were stained with PerCP-labeled anti-human CD4 (BD Biosciences) at room temperature for 30 min. After washing with FACS buffer (0.1% [w/v] BSA/PBS), the cells were stimulated with 50 ng/mL phorbol myristate acetate (PMA, Sigma-Aldrich, St. Louis, MO, USA) and 1 µg/mL ionomycin (Sigma-Aldrich) in the presence of BD GolgiStop^TM^ (BD Biosciences) for 4 h at 37 °C. The stimulated cells were washed with FACS buffer, fixed for 10 min with 4% (v/v) paraformaldehyde, permeabilized with FACS™ Perm 2 (BD Biosciences) according to the manufacturer’s instructions, and stained with FITC-conjugated anti-human IFN–γ, IL-1, IL-6 (BD Biosciences), PE-conjugated anti-human IL-10, IL-17, IFN-γ, and TNF-α (eBioscience) antibodies.

### Measurements

The following clinical data were obtained: (1) Patient demographic characteristics, including intraoperative drug dosages, fluids, and transfusion requirements; (2) changes in intraoperative haemodynamics; (3) inflammation-related laboratory tests, including white blood cell, neutrophil and lymphocyte counts, erythrocyte sedimentation rate (ESR), high-sensitivity C-reactive protein (hs-CRP); (4) levels of cardiac enzymes, including CK-MB, high-sensitivity troponin (Tn) I, and N-terminal-pro brain natriuretic peptide (NT-pro BNP); and (5) arterial blood gas analysis, including the ratio of arterial oxygen partial pressure to fractional inspired oxygen (PF ratio), and haematocrit level, potential of hydrogen (pH), and lactate level.

### Statistical analysis

The primary outcome was the difference in expression of CD39 and CD73 in circulating CD4^+^ T cells between the propofol and sevoflurane groups. The data for the sample size calculation were drawn from 10 patients per group in our pilot study using G*power (ver. 3.1.9.2; Universität Kiel, Kiel, Germany). The calculated sample size for the primary outcome was 52 in each group for CD39 and 78 in each group for CD73 from the data of our pilot study with an α of 0.05 and power of 0.8. Therefore, we included 78 patients in each group; a total of 173 patients were enrolled in our study considering a dropout rate of 10%.

An independent two-tailed *t* test was used to compare mean values in cases of continuous normally distributed data. When the data were not distributed normally, the Mann-Whitney U test was used. Intragroup changes and intergroup differences in expression levels were analysed by two way analysis of variance for repeated measurements or the Friedman test, as appropriate. If a significant difference was noted, Student’s t-test or the Mann-Whitney rank-sum test was used to compare group differences with Bonferroni’s correction applied. The chi-square test was used to compare categorical variables, and numbers of patients (n) and proportions (%) were calculated. All calculations were performed using SPSS software (ver. 20.0; IBM SPSS Inc., Chicago, IL, USA). A value of *P* < 0.05 was taken to indicate statistical significance.

## Electronic supplementary material

Supplementary Tables and Figures

## References

[1] Levy JH, Tanaka KA (2003). Inflammatory response to cardiopulmonary bypass. The Annals of thoracic surgery.

[2] De Hert S, Moerman A (2015). Myocardial injury and protection related to cardiopulmonary bypass. Best Pract Res Clin Anaesthesiol.

[3] Wan IYP (2004). Beating heart revascularization with or without cardiopulmonary bypass: evaluation of inflammatory response in a prospective randomized study. The Journal of thoracic and cardiovascular surgery.

[4] Marik PE (2005). Propofol: an immunomodulating agent. Pharmacotherapy.

[5] An, K. *et al*. Effects of propofol on pulmonary inflammatory response and dysfunction induced by cardiopulmonary bypass. *Anaesthesia* **63**, 10.1111/j.1365-2044.2008.05627.x (2008). 10.1111/j.1365-2044.2008.05627.x 18822094

[6] Lin, E. & Symons, J. A. Volatile anaesthetic myocardial protection: a review of the current literature. *HSR Proc Intensive Care Cardiovasc Anesth* **2** (2010). PMC3484611 23440181

[7] Li, F. & Yuan, Y. Meta-analysis of the cardioprotective effect of sevoflurane versus propofol during cardiac surgery. *BMC Anesthesiol* **15**, 10.1186/s12871-015-0107-8 (2015). 10.1186/s12871-015-0107-8 PMC4583176 26404434

[8] Pasin L (2015). Propofol and survival: a meta-analysis of randomized clinical trials. Acta anaesthesiologica Scandinavica.

[9] Antonioli L, Pacher P, Vizi ES, Hasko G (2013). CD39 and CD73 in immunity and inflammation. Trends Mol Med.

[10] Antonioli, L. *et al*. Adenosine and inflammation: what’s new on the horizon? *Drug Discov Today*, 10.1016/j.drudis.2014.02.010 (2014). 10.1016/j.drudis.2014.02.010 24607729

[11] Eckle T (2007). Cardioprotection by ecto-5′-nucleotidase (CD73) and A2B adenosine receptors. Circulation.

[12] Kohler D (2007). CD39/ectonucleoside triphosphate diphosphohydrolase 1 provides myocardial protection during cardiac ischemia/reperfusion injury. Circulation.

[13] Roberts V, Lu B, Rajakumar S, Cowan PJ, Dwyer KM (2013). The CD39-adenosinergic axis in the pathogenesis of renal ischemia-reperfusion injury. Purinergic Signal.

[14] Sung SSJ (2017). Proximal tubule CD73 Is Critical in renal ischemia-reperfusion injury protection. J. Am. Soc. Nephrol..

[15] Kim M (2013). The volatile anesthetic isoflurane induces ecto-5′-nucleotidase (CD73) to protect against renal ischemia and reperfusion injury. Kidney Int.

[16] Bonner F, Borg N, Burghoff S, Schrader J (2012). Resident cardiac immune cells and expression of the ectonucleotidase enzymes CD39 and CD73 after ischemic injury. PLoS One.

[17] Blume C (2012). Autoimmunity in CD73/Ecto-5′-nucleotidase deficient mice induces renal injury. PLoS One.

[18] Kinsey GR (2012). Autocrine adenosine signaling promotes regulatory T cell-mediated renal protection. J Am Soc Nephrol.

[19] Yang X-L, Wang D, Zhang G-Y, Guo X-L (2017). Comparison of the myocardial protective effect of sevoflurane versus propofol in patients undergoing heart valve replacement surgery with cardiopulmonary bypass. BMC Anesthesiology.

[20] Likhvantsev, V. V. *et al*. Sevoflurane versus total intravenous anesthesia for isolated coronary artery bypass surgery with cardiopulmonary bypass: a randomized trial. *Journal of cardiothoracic and vascular anesthesia* **30**, 10.1053/j.jvca.2016.02.030 (2016). 10.1053/j.jvca.2016.02.030 27431595

[21] Bignami E (2009). Role of cardiac biomarkers (troponin I and CK-MB) as predictors of quality of life and long-term outcome after cardiac surgery. Ann Card Anaesth.

[22] Bettex, D. A. *et al*. Role of sevoflurane in organ protection during cardiac surgery in children: a randomized controlled trial. *Interact Cardiovasc Thorac Surg* **20**, 10.1093/icvts/ivu381 (2015). 10.1093/icvts/ivu381 25404229

[23] Desborough J (2000). The stress response to trauma and surgery. British journal of anaesthesia.

[24] Cardinale F (2011). Perioperative period: immunological modifications. Int J Immunopathol Pharmacol.

[25] Iwagaki H (2001). Blood transfusion and postoperative plasma cytokine antagonist levels in colorectal cancer patients. Hepato-gastroenterology.

[26] Lurati Buse GA (2012). Randomized comparison of sevoflurane versus propofol to reduce perioperative myocardial ischemia in patients undergoing noncardiac surgery. Circulation.

[27] Covarrubias R (2016). Role of the CD39/CD73 Purinergic Pathway in Modulating Arterial Thrombosis in Mice. Arteriosclerosis, Thrombosis, and Vascular Biology.

[28] Bono MR, Fernandez D, Flores-Santibanez F, Rosemblatt M, Sauma D (2015). CD73 and CD39 ectonucleotidases in T cell differentiation: Beyond immunosuppression. FEBS Lett.

[29] Wheeler DG (2012). Transgenic swine: expression of human CD39 protects against myocardial injury. Journal of molecular and cellular cardiology.

[30] Crikis S (2010). Transgenic overexpression of CD39 protects against renal ischemia-reperfusion and transplant vascular injury. Am J Transplant.

[31] Ramakers BP (2008). Measurement of the endogenous adenosine concentration in humans *in vivo*: methodological considerations. Current drug metabolism.

[32] Regateiro FS, Cobbold SP, Waldmann H (2013). CD73 and adenosine generation in the creation of regulatory microenvironments. Clinical and experimental immunology.

[33] Kang WS, Yoon TG, Kim TY, Kim SH (2011). Comparison of the PaO2/FiO2 ratio in sternotomy vs. thoracotomy in mitral valve repair: a randomised controlled trial. European journal of anaesthesiology.

