# Supplementary material for: Comparison of the expression of cluster of differentiation (CD)39 and CD73 between propofol- and sevoflurane-based anaesthesia during open heart surgery
Source: Sci Rep. 2018 Jul 5;8:10197. doi: 10.1038/s41598-018-28505-8 (PMC6033940; doi:10.1038/s41598-018-28505-8)
Supplement: Supplementary file 1 — Supplementary Tables and Figures [file 41598_2018_28505_MOESM1_ESM.docx]

***Supplemetary Table 1.*** *The mean fluorescence intensities of CD39 and CD73 in helper T cells and regulatory T cells.*

|  |  | Propofol group  (n = 78) | Sevoflurane group (n = 78) | *P* |
| --- | --- | --- | --- | --- |
| CD39 in CD4^+^ T cells | | |  |  |
|  | Preop | 413.5 (315.0 - 546.0) | 449.5 (296.0 - 459.0) | 0.154 |
|  | Weaning | 466.5 (274.0 - 496.0) | 408.0 (363.0 - 416.0) | 0.518 |
|  | 3 hours | 392.5 (312.0 - 445.0) | 334.0 (287.0 - 357.0) | < 0.001 |
|  | 24 hours | 440.5 (268.0 - 518.0) | 327.0 (311.0 - 352.0) | 0.014 |
|  | 48 hours | 400.0 (273.0 - 497.0) | 419.0 (395.0 - 435.0) | 0.296 |
| CD73 in CD4^+^ T cells | | |  |  |
|  | Preop | 562.0 (517.0 - 615.0) | 564.0 (524.0 - 702.0) | 0.825 |
|  | Weaning | 565.0 (527.0 - 587.0) | 518.5 (494.0 - 588.0) | < 0.001 |
|  | 3 hours | 662.0 (603.0 - 697.0) | 593.0 (565.0 - 632.0) | < 0.001 |
|  | 24 hours | 789.0 (732.0 - 819.0) | 700.0 (673.0 - 787.0) | < 0.001 |
|  | 48 hours | 762.0 (649.0 - 807.0) | 717.0 (648.0 - 772.0) | 0.140 |
| CD39 in CD4^+^CD25^+^Foxp3^+^ T cells | | |  |  |
|  | Preop | 1207.5 (881.0 - 1745.0) | 1385.5 (1125.0 - 1580.0) | 0.119 |
|  | Weaning | 1215.0 (882.0 - 1534.0) | 1053.0 (1010.0 - 1365.0) | 0.149 |
|  | 3 hours | 999.5 (806.0 - 1316.0) | 999.0 (851.0 - 1319.0) | 0.814 |
|  | 24 hours | 953.5 (643.0 - 1244.0) | 885.0 (794.0 - 1017.0) | 0.770 |
|  | 48 hours | 910.0 (642.0 - 1848.0) | 1048.5 (899.0 - 1204.0) | 0.227 |
| CD39 in CD4^+^CD25^+^Foxp3^+^ T cells | | |  |  |
|  | Preop | 1174.5 (845.0 - 1469.0) | 1206.5 (998.0 - 1283.0) | 0.597 |
|  | Weaning | 1103.0 (841.0 - 1491.0) | 1142.0 (814.0 - 1262.0) | 0.462 |
|  | 3 hours | 1127.0 (872.0 - 1343.0) | 1122.5 (971.0 - 1240.0) | 0.999 |
|  | 24 hours | 1220.0 (656.0 - 1862.0) | 1236.0 (846.0 - 1398.0) | 0.942 |
|  | 48 hours | 1196.0 (294.0 - 1913.0) | 1144.0 (520.0 - 1515.0) | 0.816 |
|  |  |  |  |  |

Data are expressed as median values (25 – 75%).

**Abbreviations:** CD, cluster of differentiation; Preop, preoperative time; Weaning, immediate after weaning from cardiopulmonary bypass (CPB); 3 hours, 3 hours after weaning from CPB; 24 hours, 24 hours after weaning from CPB; 48 hours, 48 hours after weaning from CPB.

***Supplementary figures***

***Supplementary Figure 1.*** *The expression of CD39 in circulating helper T cells during open heart surgery (OHS).*

The expression of CD39 was lowest 3 hours after weaning from cardiopulmonary bypass (CPB). ^*^Comparison (*P* < 0.05) to Preop. ^†^Comparison (*P* < 0.05) to Weaning. ^‡^Comparison (*P* < 0.05) to 3 hours. ^§^Comparison (*P* < 0.05) to 24 hours.


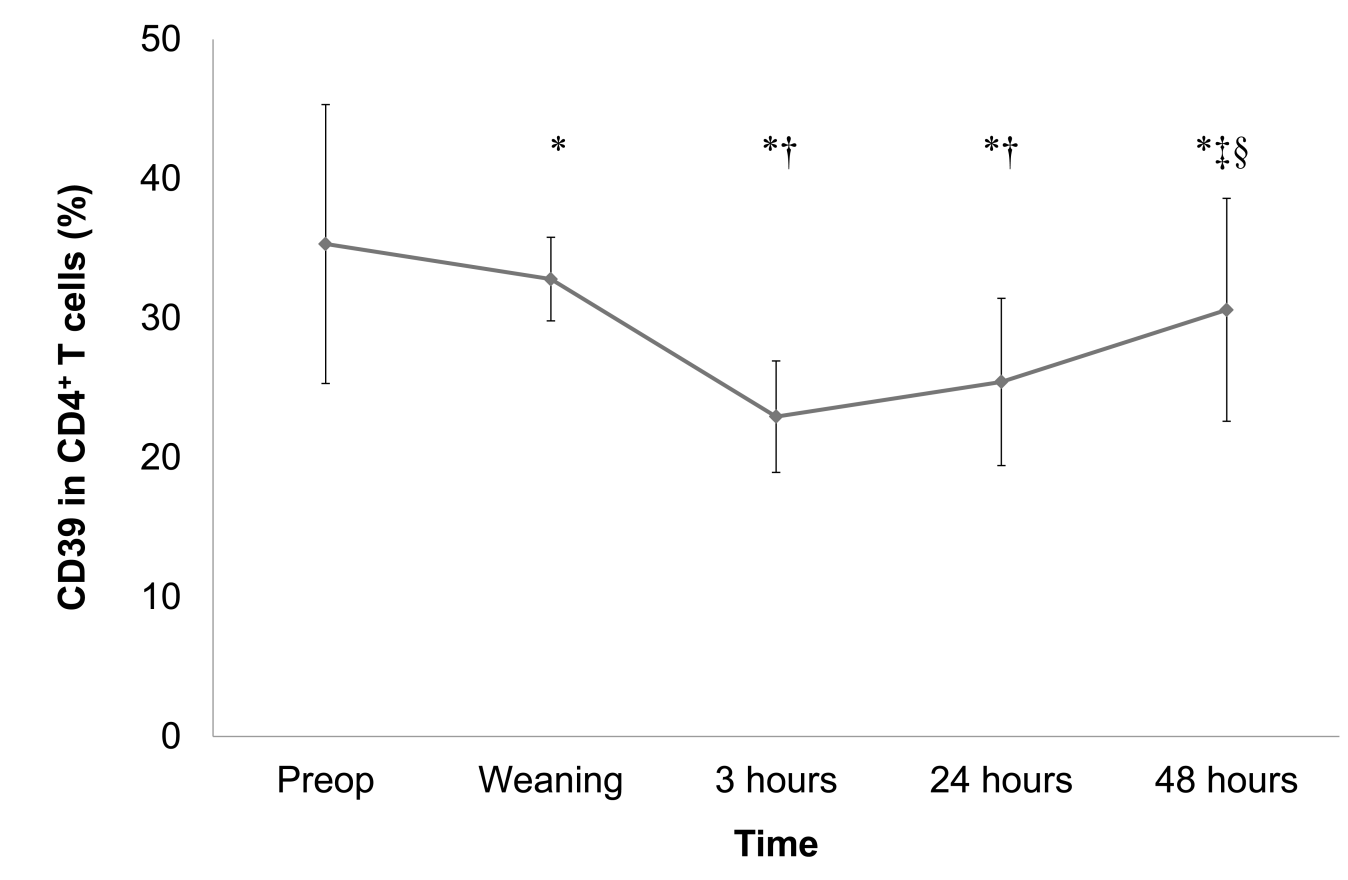


**Abbreviations:** Preop, preoperative time; Weaning, immediate after weaning from cardiopulmonary bypass (CPB); 3 hours, 3 hours after weaning from CPB; 24 hours, 24 hours after weaning from CPB; 48 hours, 48 hours after weaning from CPB.

***Supplementary Figure 2.*** *The expression of CD73 in circulating helper T cells during OHS.*

The expression of CD39 was lowest immediately after weaning from CPB. ^*^Comparison (*P* < 0.05) to Preop. ^†^Comparison (*P* < 0.05) to Weaning. ^‡^Comparison (*P* < 0.05) to 3 hours. ^§^Comparison (*P* < 0.05) to 24 hours.


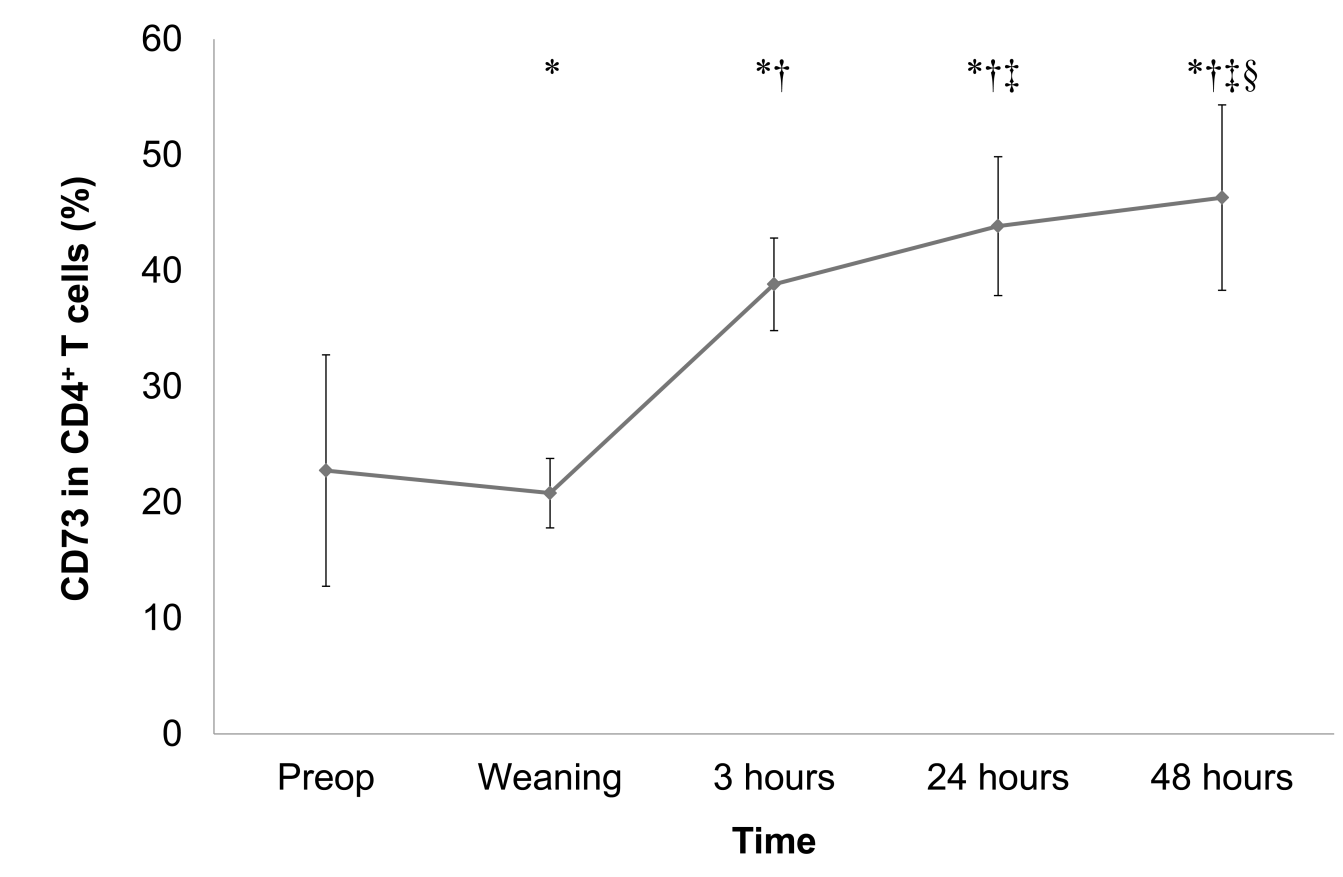


**Abbreviations:** Preop, preoperative time; Weaning, immediate after weaning from CPB; 3 hours, 3 hours after weaning from CPB; 24 hours, 24 hours after weaning from CPB; 48 hours, 48 hours after weaning from CPB.

***Supplementary Figure 3.*** *The expression of CD39 in circulating regulatory T cells during OHS.*

The expression of CD39 was lowest 3 hours after weaning from CPB. ^*^Comparison (*P* < 0.05) to Preop. ^†^Comparison (*P* < 0.05) to Weaning. ^‡^Comparison (*P* < 0.05) to 3 hours. ^§^Comparison (*P* < 0.05) to 24 hours.


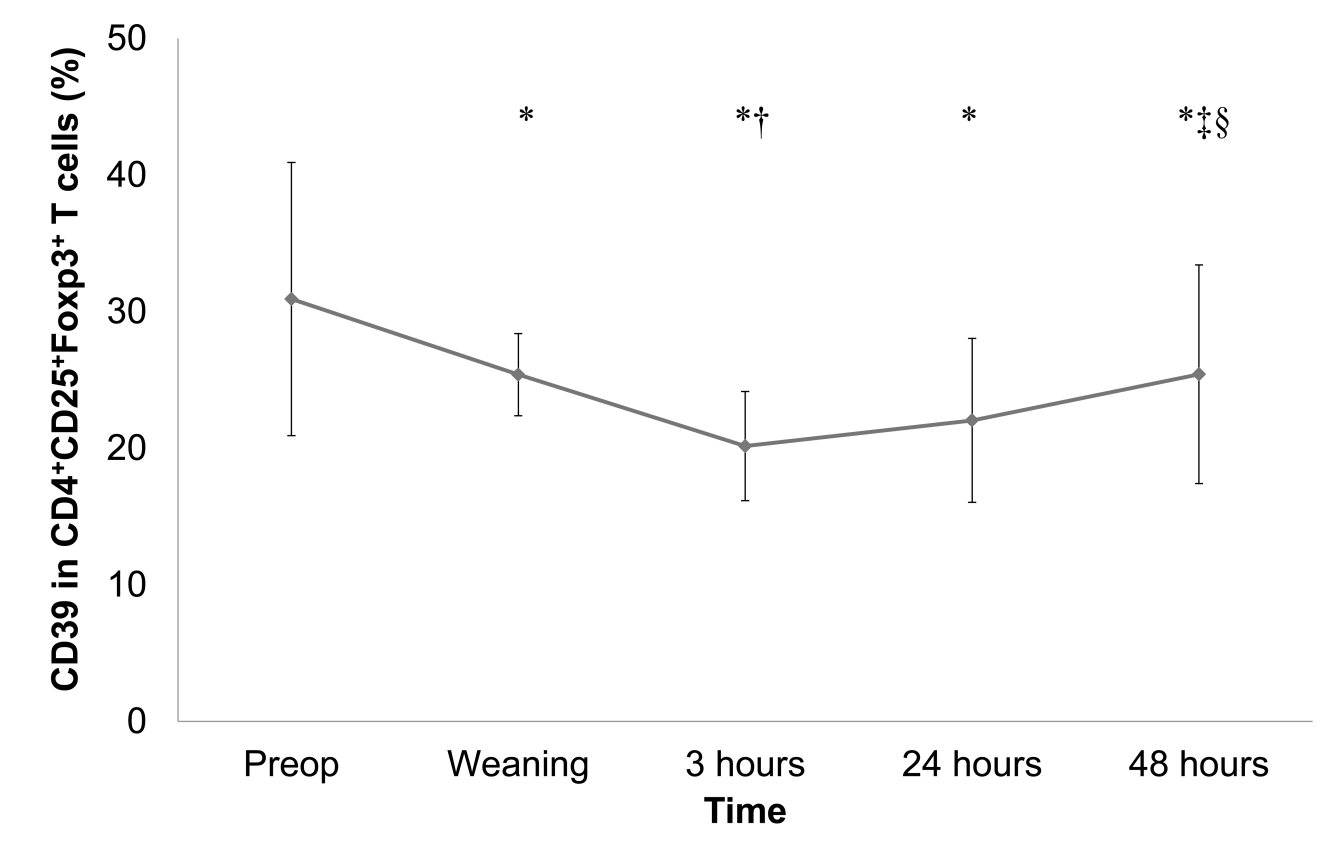


**Abbreviations:** Preop, preoperative time; Weaning, immediate after weaning from CPB; 3 hours, 3 hours after weaning from CPB; 24 hours, 24 hours after weaning from CPB; 48 hours, 48 hours after weaning from CPB.

***Supplementary Figure 4.*** *The expression of CD73 in circulating regulatory T cells during OHS.*

The expression of CD73 was lowest immediately after weaning from CPB. ^*^Comparison (*P* < 0.05) to Preop. ^†^Comparison (*P* < 0.05) to Weaning. ^‡^Comparison (*P* < 0.05) to 3 hours.


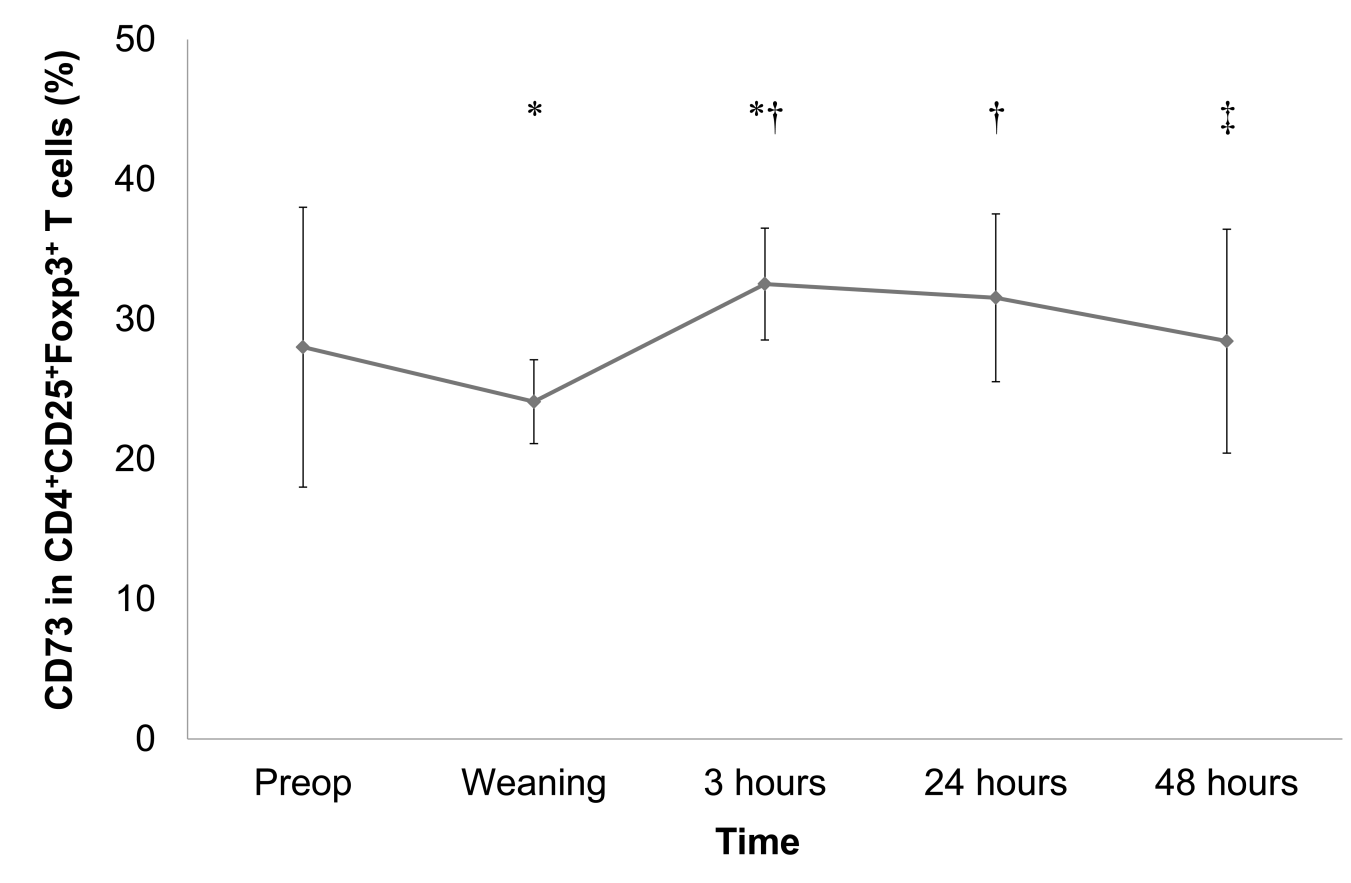


**Abbreviations:** Preop, preoperative time; Weaning, immediate after weaning from CPB; 3 hours, 3 hours after weaning from CPB; 24 hours, 24 hours after weaning from CPB; 48 hours, 48 hours after weaning from CPB.

***Supplementary Figure 5.*** *Comparison of changes in CD39 expression in circulating regulatory T cells between the propofol- and sevoflurane-based anaesthesia groups during OHS.*

The overall expression of CD39 was lower in the sevoflurane group. However, statistical significant difference was not detected between the two groups*.*


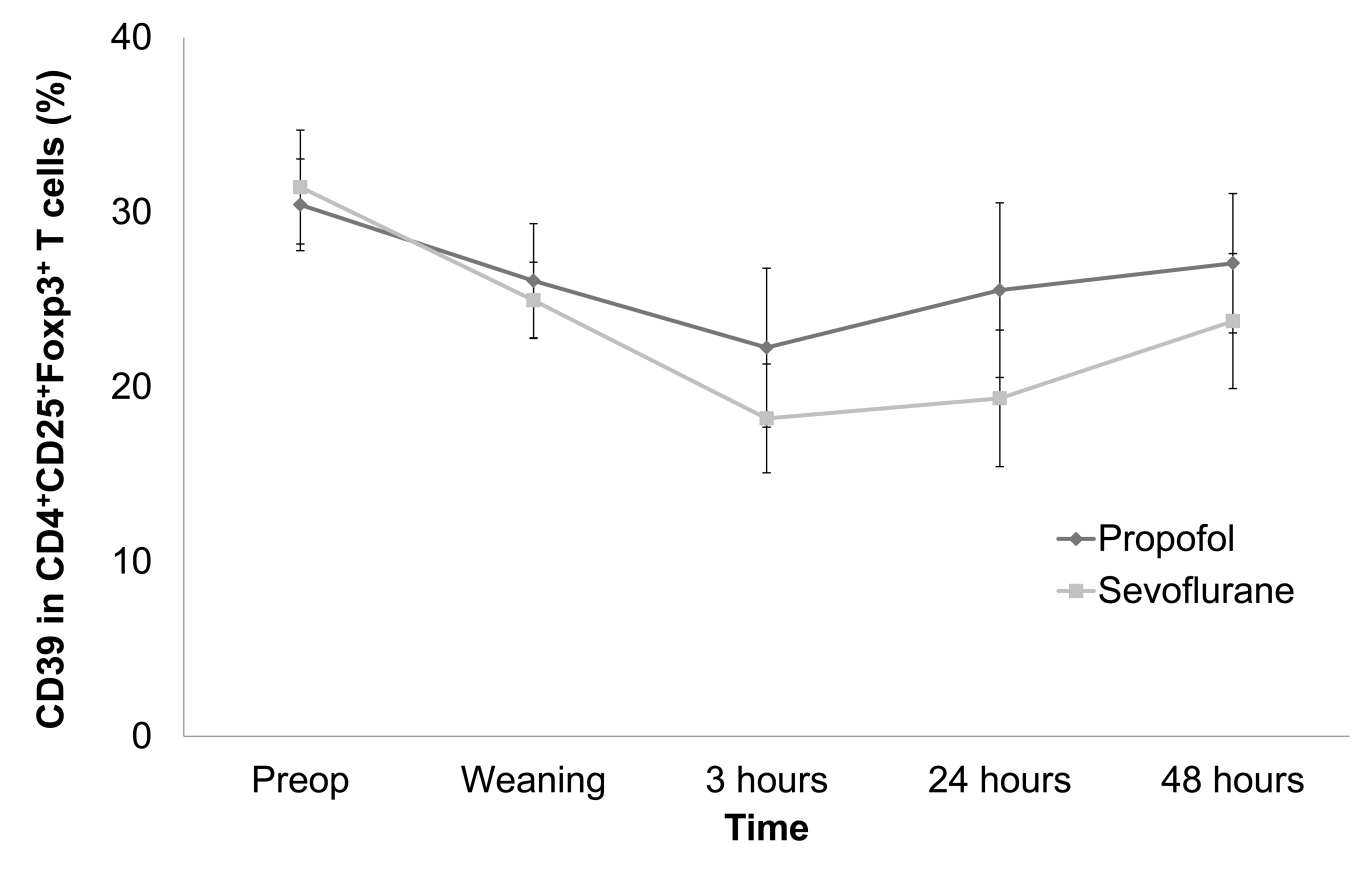


**Abbreviations:** Preop, preoperative time; Weaning, immediate after weaning from CPB; 3 hours, 3 hours after weaning from CPB; 24 hours, 24 hours after weaning from CPB; 48 hours, 48 hours after weaning from CPB.

***Supplementary Figure 6.*** *Comparison of changes in CD73 expression in circulating regulatory T cells between the propofol- and sevoflurane-based anaesthesia groups during OHS.*

The overall expression of CD39 was lower in the sevoflurane group. However, statistical significant difference was not detected between the two groups*.*


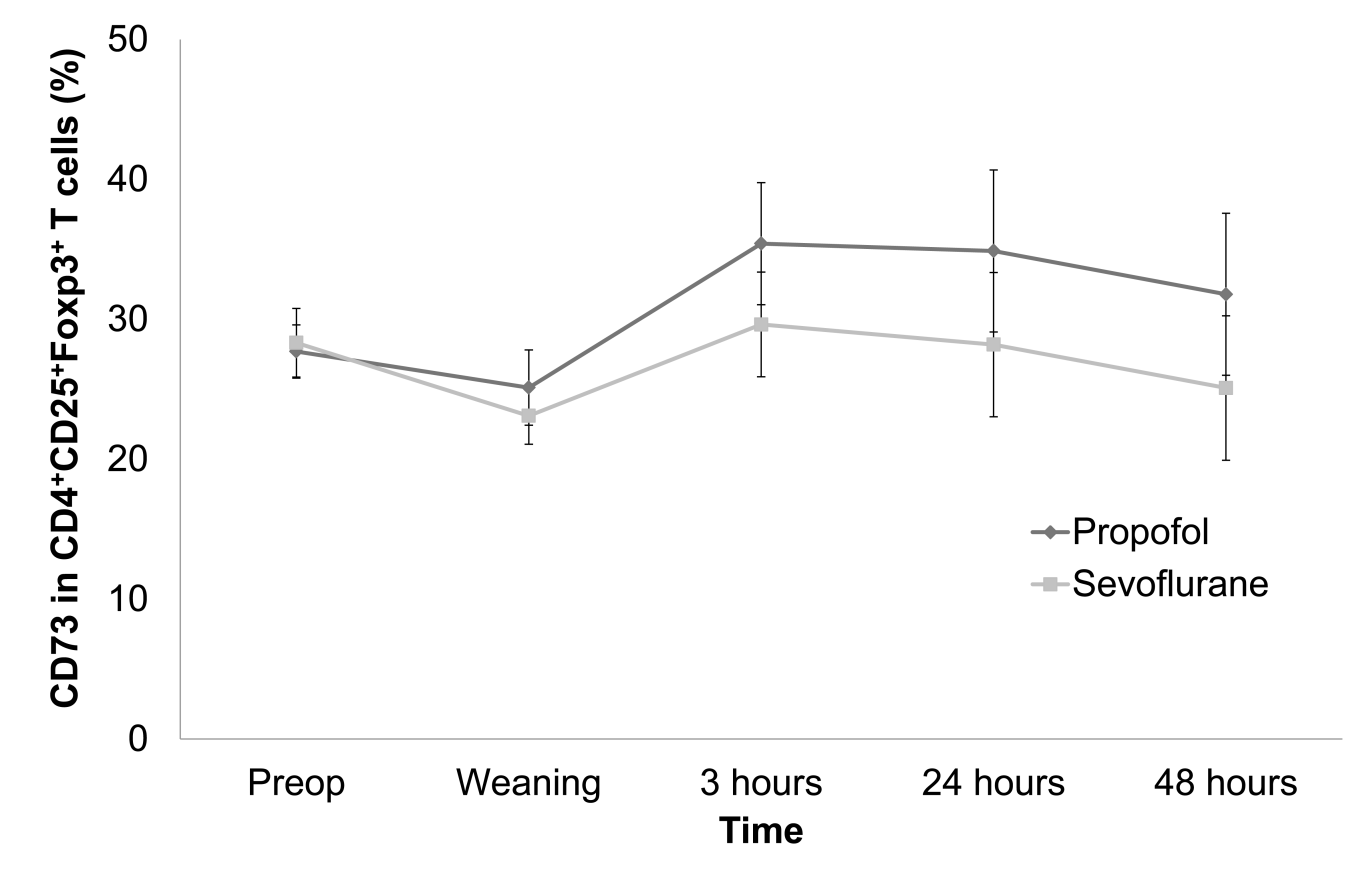


**Abbreviations:** Preop, preoperative time; Weaning, immediate after weaning from CPB; 3 hours, 3 hours after weaning from CPB; 24 hours, 24 hours after weaning from CPB; 48 hours, 48 hours after weaning from CPB.
